# Megakaryocytic Potentiating Factor and Mature Mesothelin Stimulate the Growth of a Lung Cancer Cell Line in the Peritoneal Cavity of Mice

**DOI:** 10.1371/journal.pone.0104388

**Published:** 2014-08-13

**Authors:** Jingli Zhang, Tapan K. Bera, Wenhai Liu, Xing Du, Christine Alewine, Raffit Hassan, Ira Pastan

**Affiliations:** Laboratory of Molecular Biology, Center for Cancer Research, National Cancer Institute, National Institutes of Health, Bethesda, Maryland, United States of America; Memorial Sloan-Kettering Cancer Center, United States of America

## Abstract

The mesothelin (*MSLN*) gene encodes a 71 kilodalton (kDa) precursor protein that is processed into megakaryocytic potentiating factor (MPF), a 31 kDa protein that is secreted from the cell, and mature mesothelin (mMSLN), a 40 kDa cell surface protein. The mMSLN binds to CA125, an interaction that has been implicated in the intra-cavitary spread of mesothelioma and ovarian cancer. To better define the role of MPF and mMSLN, growth of the lung cancer cell line A549 was evaluated in immuno-deficient mice with inactivation of the *Msln* gene. We observed that *Msln*–/– mice xenografted with intraperitoneal A549 tumors survive significantly long than tumor-bearing *Msln+/+* mice. When tumor-bearing *Msln*–/– *mice* are supplemented with recombinant MPF (and to a lesser extent mMSLN), most of this survival advantage is lost. These studies demonstrate that MPF and mMSLN have an important role in the growth of lung cancer cells *in* *vivo* and raise the possibility that inactivation of MPF may be a useful treatment for lung and other MSLN expressing cancers.

## Introduction

**Figure 1:**
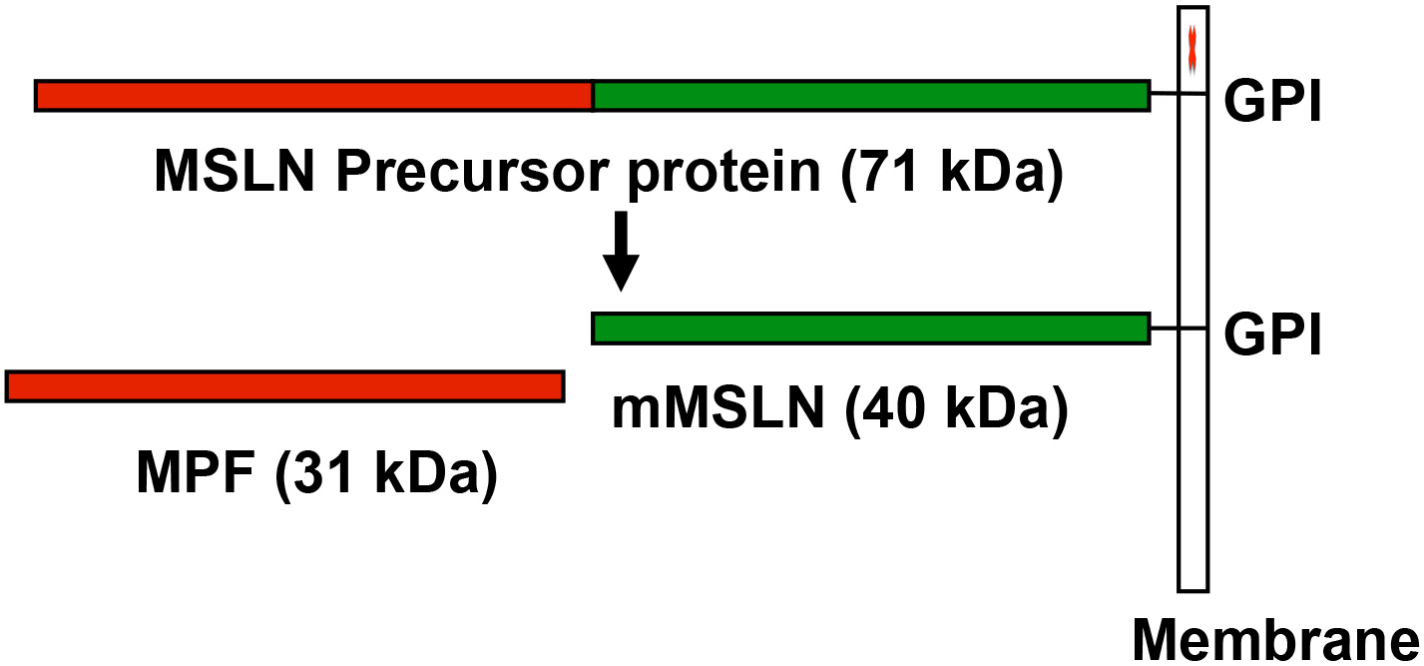
Schematic showing MSLN precursor protein, which is cleaved to MPF and mMSLN. GPI, glycosylphosphatidylinositol.

The mesothelin *(MSLN)* gene encodes a lineage restricted tumor antigen that is expressed in normal mesothelial cells lining the pleura, peritoneum and pericardium. It is also highly expressed in epitheliod mesotheliomas, which are derived from normal mesothelial cells, and aberrantly in many other cancers including ovarian, pancreatic, lung, stomach, cholangiocarcinoma and triple negative breast cancer [1]–[4]. The *MSLN* gene encodes a 71 kDa precursor protein that is processed into two mature proteins: Megakaryocytic Potentiating Factor (MPF) and mature MSLN (mMSLN). MPF is a 31 kDa glycoprotein that is secreted from the cell into the blood. The mMSLN is a 40 kDa glycoprotein protein that remains bound to the cell membrane by phosphatidyl inositol [5], [6], but sheds from the cell surface over time via the action of TNF-α converting enzyme (Fig. 1) [7]. The mMSLN has been shown to bind to MUC16 (CA125); this interaction has been implicated in the intra-cavitary spread of mesothelioma and ovarian cancer [8]. Studies of *Msln* gene knock out(–/–) mice indicate that the gene is not essential for normal development and reproduction, but several recent studies have raised the possibility that *MSLN* might regulate cancer cell growth [9]–[12]. In Eker rats, the development of tuberous sclerosis-2-induced renal carcinoma was significantly reduced in the absence of a homologue of the *MSLN* gene [13], [14]. In pancreatic cancer cells, over expression of *MSLN* has been implicated in significant enhancement of tumor cell growth and migration *in* *vitro* [11]. Recently the expression level of MSLN protein was correlated with tumor aggressiveness as well as decreased overall survival of patients with early-stage lung adenocarcinoma [15], but the molecular mechanism that contributes to these phenotypes are not well understood. CA125, a membrane associated ovarian cancer antigen, has been reported to interact with MSLN, and it has been suggested that this interaction may facilitate ovarian cancer metastasis. Although binding of MSLN-expressing cells to CA125 has been demonstrated in cell culture [8], [16], there are no animal experiments showing that this interaction is important in tumor bearing mice.

We have generated athymic nude mice in which the *Msln* gene is inactivated, preventing the production of mMsln and Mpf protein. These mice reproduce normally and no abnormalities in their growth or other properties have been observed. Because these mice do not make Mpf and do not have Msln on the cells that line the peritoneal cavity, we have used them to investigate whether the absence of Msln affects the growth of lung cancer cells inoculated within the peritoneal cavity of these mice.

## Materials and Methods

### Cell Lines and Immunohistochemistry

The lung cancer cell line A549 was obtained from Dr. Hisataka Kobayashi (National Cancer Institute, NIH, Bethesda, USA) and its identity was confirmed by DNA fingerprinting using Short Tandem Repeats assay. The human epidermoid carcinoma cell line A431 was purchased from ATCC (Mananasas, VA) and maintained in the laboratory as recommended. Immunohistochemical staining for MSLN and CA125 was performed by Histoserv, Inc (Germantown, MD) using mouse anti-MSLN 5B2 (Novocastra Reagents from Leica Bioystems, Buffalo Grove, IL) as previously described [17], and mouse anti-CA125 OC125 (Zymed Laboratories) at 1:50 dilution.

### Animal Experiments

The Animal Study Proposals (LMB-053 and LMB-014) were approved by the NIH Animal Care and Use Committee. All animal experiments including treatment and sacrifice were conducted according to the institutional guidelines of the NIH. Athymic nude mice (NCr-nu/nu) and Nude/*Msln*–/– mice obtained from NCI-Frederick were housed in micro-isolator cages throughout the course of the experiment. All mice used for experiments were females, weight (15–20 g) and age (7–10 weeks) matched. All injections were intraperitoneal (IP) unless otherwise indicated. A549 and A431 IP xenografts were established by inoculating 2×10^6^ cells in 200 µl of cell suspension in phosphate buffered saline (PBS). For the subcutaneous model, 2×10^6^ A549 cells were injected into the right thigh. All recombinant protein treatments were performed by IP injection of 25 µg study protein in 200 µl PBS, three times weekly for a total of three weeks. For survival studies, mice were monitored daily and sacrificed by CO_2_ inhalation when moribund.

### Generation of *Msln*–/– Athymic Nude Mice

Male *Msln* null mice in C57/Bl6 genetic background (*Msln*–/–) [9] were used to back cross with female BALB/c wild type (WT) mice and litters were screened for *Msln* heterozygous allele. Male heterozygote mice from each generation were used to back cross with WT BALB/c females and back crossing was performed for more than ten generations. Males heterozygous for *Msln* in BALB/c genetic background were crossed with athymic nude (Fox N) females to generate Fox N null/*Msln*–/– mice. Since Fox N null/*Msln*–/– female mice are not good breeders, we used *Msln*–/–/Fox N Het females as the breeding partners for Fox N null/*Msln*–/– males to maintain and generate Fox N null/*Msln*–/– females for our experiments.

### Generation of MPF-Rabbit(r)Fc, mMSLN-rFc and CD22-rFc Fusion Proteins in Mammalian Cells

To generate recombinant versions of mMSLN and MPF, mMSLN-rFc and MPF-rFc were expressed as fusion proteins with rabbit IgG (rFc) in HEK293T cells and purified as previously described [18]. CD22-rFc fusion protein was prepared for use as a control.

### Flow Cytometry

Cells were harvested, washed and re-suspended in ice-cold FACS buffer (PBS with 5% FBS and 0.1% sodium azide), then incubated with anti-mesothelin antibody, MN (Rockland Immunochemicals Inc., Gilbertsville, PA) or Morab-009 (Morphotek Inc, Exton, PA) or anti-MUC16 antibody, OC125 (Abcam, Cambridge, MA) at concentration of 5 µg/ml. Isotope-matched antibody was used as a control. The binding of primary antibodies to tumor cells was detected by using either goat anti-mouse conjugated with R-phycoerythrin (R-PE) (Invitrogen) or with goat anti-mouse conjugated with FITC (Biosource). The fluorescence associated with the live cells was determined using a FACS Calibur (BD Biosicences). Geometric means were expressed as mean fluorescence intensity (MFI). The MFI of cells was compared with the MFI from a standard curve of R-PE-conjugated calibration beads (BD QuantiBRITETM PE quantitation kit, BD Biosciences) and the number of mMSLN sites per cell was estimated. Results were similar with MN (n = 2 experiments) or Morab-009 (n = 3 experiments).

### MPF Quantification

A549 cells stably transfected with either vector control (A549/pcDNA) or MPF expression vector (A549/MPF) were plated into dishes containing equal amount of complete medium and incubated for 48 hours. Total cell population was counted using a Cellometer Vision cell counter (Nexcelom, St. Lawrence, MA) An equivolume aliquot of conditioned medium was removed from each dish and the concentration of MPF in the solution was quantified using a proprietary human MPF enzyme-linked immunosorbent assay kit (Morphotek, Easton, PA).

### Soft Agar Assay

A549/pcDNA or A549/MPF stable transfectants were suspended in 0.35% agar, plated in 6 well plates (2×10^3^ cells/well), and incubated for 15–21 days at 37°C. After Crystal Violet staining the plates were washed for 1 hour then scanned to produce digital images. Colonies were counted within a pre-specified, centrally-placed square field with side length equal to one-third of the well diameter. Multiple wells (n) were counted for each of three independent experiments. The mean number of colonies for each experiment are reported with standard deviation (SD). KB cells were used as a positive control for colony formation.

### Statistical Analysis

For survival studies, Kaplan-Meier curves were plotted and compared using the log-rank test. A Mann-Whitney test was used for statistical comparison of survival data. P<0.05 was used as the threshold for statistical significance. All statistical analysis was performed using Prism (version 5) for Mac (GraphPad software).

## Results

**Figure 2:**
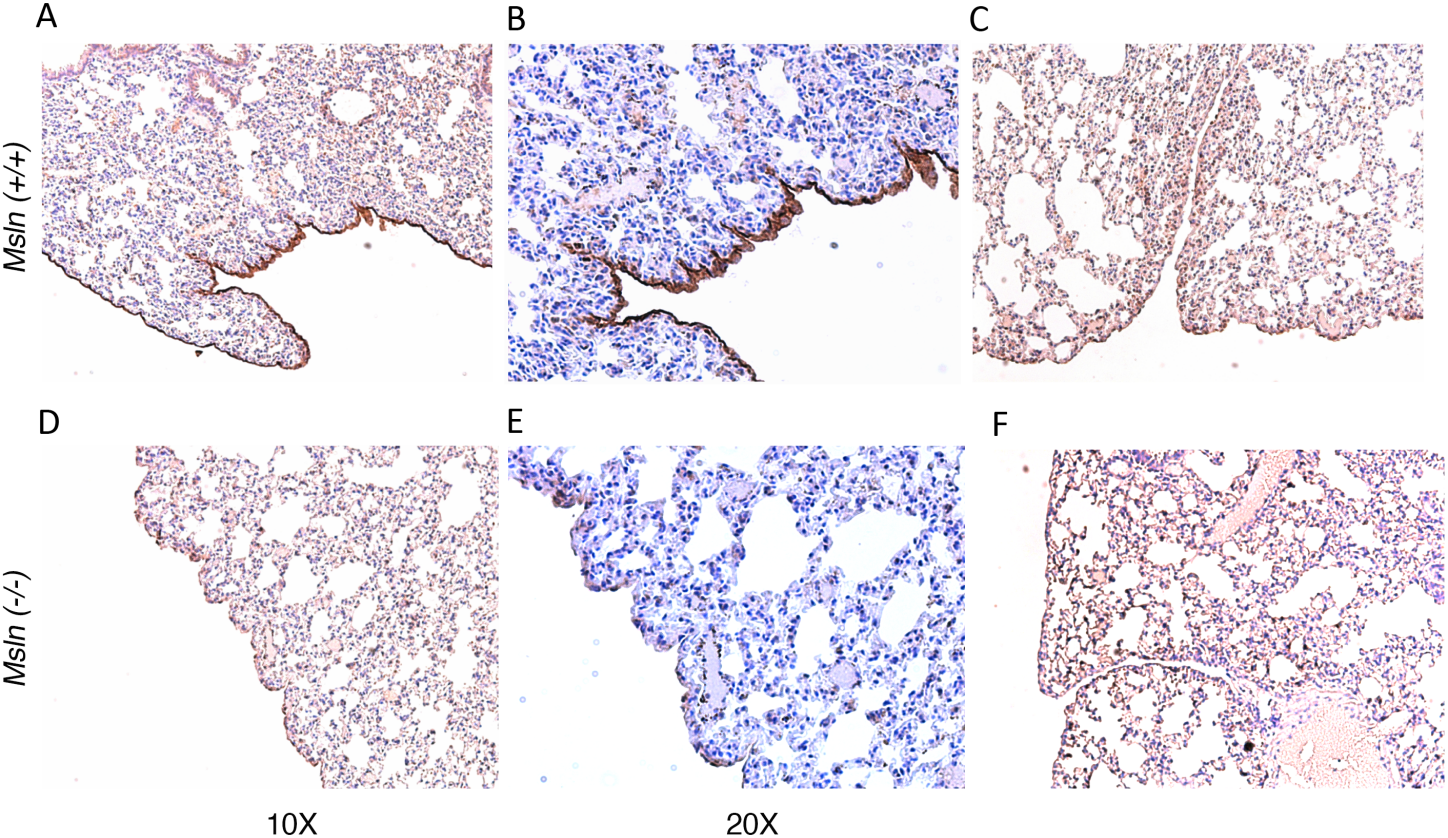
Immunohistochemical analysis of mesothelin protein in lung tissue from *FoxN null/Msln*(+/+) and *FoxN null/Msln*(–/–) mice. A portion of lung tissues were fixed in 4% paraformaldehyde embedded in paraffin and sectioned 5 to 6 µm in thickness, then stained for MSLN. **A–C:** Lung tissue form *FoxN null/Msln*(+/+) stained with anti-Msln antibody (A: 100X; B: 200X magnification); or secondary antibody only (C: 100X magnification) as negative control. **D–F:** Lung tissue form *FoxN null/Msln*(–/–) stained with anti-Msln antibody (D: 100X; E: 200X magnification); or secondary antibody only (F: 100X magnification) as negative control. As expected strong mesothelin expression was detected in the mesothelial cell lining of the lung from *FoxN null/Msln*(+/+) mice but no expression in lung from *FoxN null/Msln*(–/–) mice.

**Figure 3:**
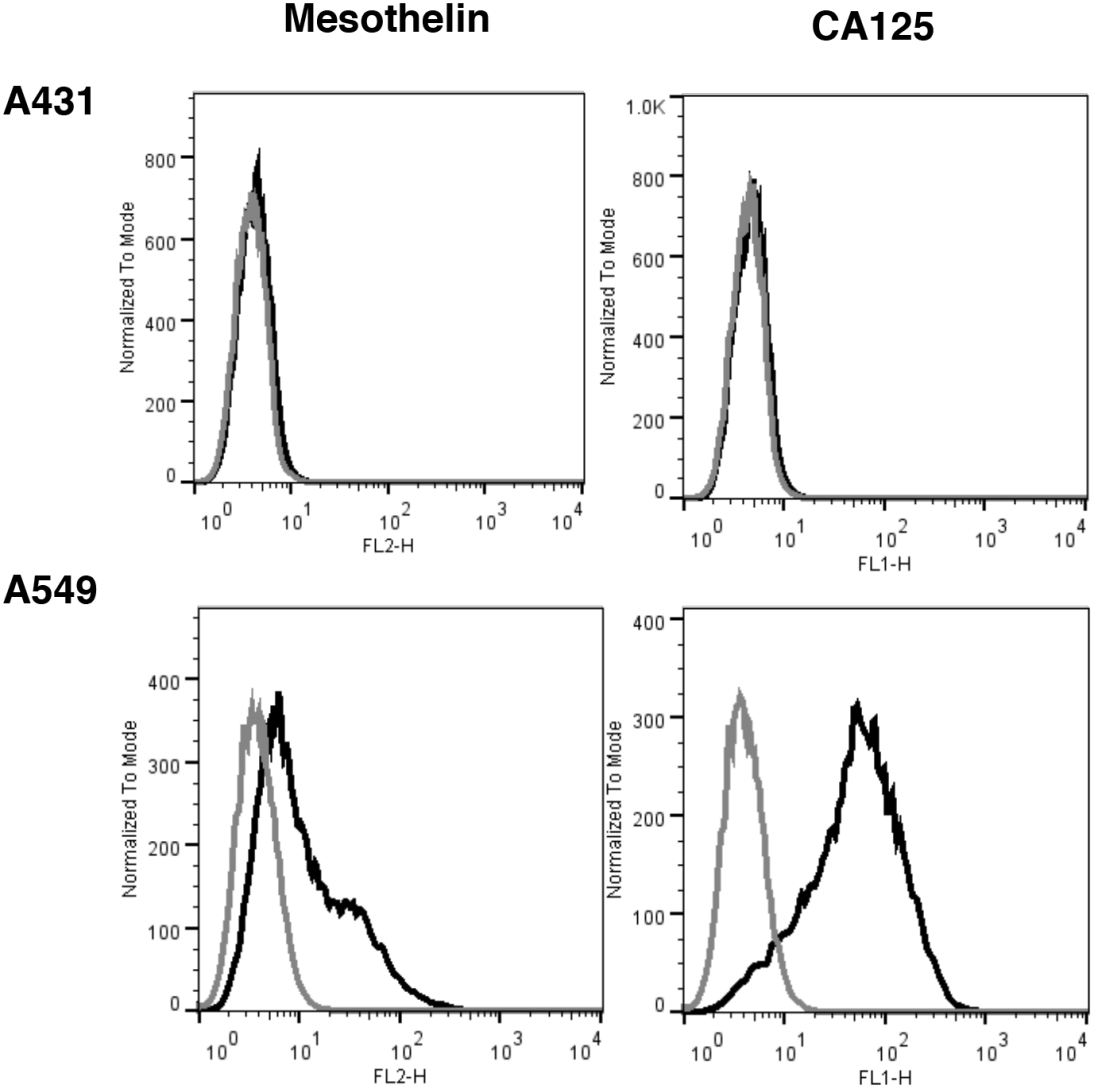
*FACS* analysis for mesothelin and CA125 expressions in A431 and A549 cell lines. Cells were incubated with the indicated primary antibodies or isotope control antibodies, and appropriate secondary antibody (goat anti-mouse IgG, R-PE or goat anti-mouse, FITC). Results are shown as histogram plots for binding of primary antibody (black trace) or isotype control (gray trace). A431 cells are negative for both MSLN and CA125. A549 cells show low mesothelin expression (3.5×10^3^ sites/cell) but highly express CA125.

**Figure 4:**
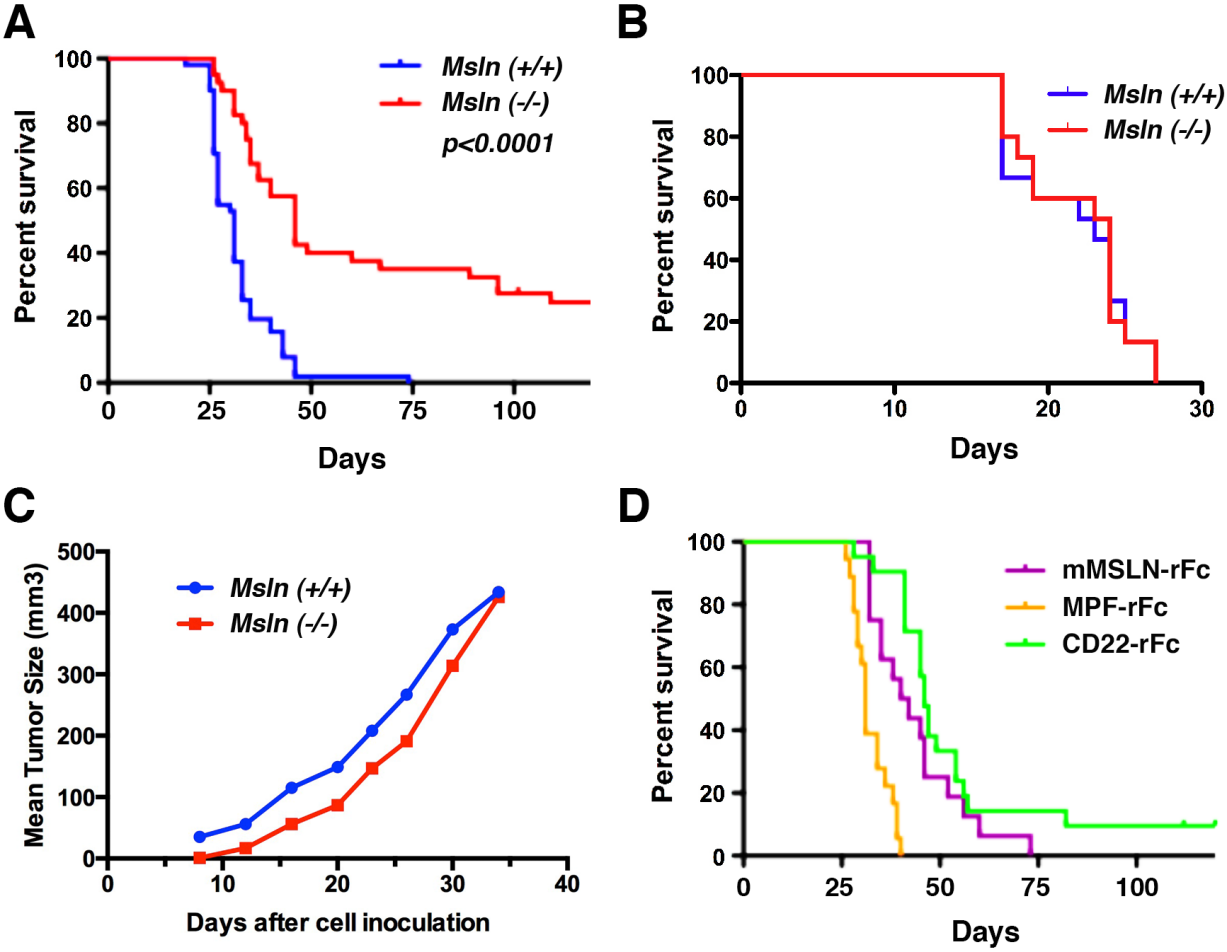
Kaplan-Meier curve showing survival of *Msln*(+/+) and *Msln*(–/–) mice bearing A549 or A431 xenografts. (A) Plot showing survival of mice after receiving IP injection of A549 cells; median survival of *Msln*–/– mice is 46 days and of *Msln(+/+)* mice is 31 days (*p*<0.0001). (B) Plot showing survival of *Msln*(–*/*–) or *Msln*(+/+) mice after IP injection of A431 cells. (C) Plot showing survival of *Msln*(–*/*–) or *Msln(+/+)* mice after subcutaneous injection of A549 cells (D) Survival of A549 xenograft bearing *Msln*(–/–) mice after receiving MPF-rFc or mMSLN-rFc or CD22-rFc proteins.

Previous studies have shown that mMSLN interacts with the ovarian cancer tumor antigen CA125 and that this interaction may be important in metastatic spread of ovarian cancer through the peritoneal cavity [8], [16], [19]. To assess the impact of MSLN on IP growth of human cancer cells, we created Fox N null/*Msln*–/– mice. To ensure that the *Msln* gene was inactivated and that the mice did not make Msln we first confirmed the deletion using polymerase chain reaction to screen for the deleted alleles of the *Msln* gene (data not shown). Lack of mMsln protein expression was confirmed by immunohistochemical analysis of mouse tissues using rabbit polyclonal anti-Msln antibody followed by color detection as previously described [9] (Fig. 2). The immune suppression caused by the FoxN deletion allows for xenograft experiment with human tumor cells. We used the lung cancer cell line A549, which has very low expression of MSLN (average of 3.5×10^3^ mMSLN binding sites/cell) but robust expression of CA125 (Fig. 3). We injected two million A549 cells into the peritoneal cavity of *Msln*–/– and *Msln+/+* nude mice, then monitored the survival of the animals. As shown in Figure 4A, there was a statistically significant difference in survival between the two groups of mice. The median survival of *Msln+/+* mice was 31 days, but increased to 46 days in *Msln*–/– mice (p<0.0001). Nearly 25% of the *Msln*–/– mice survived over four months (Table 1), while all of the *Msln+/+* mice died within 50 days (Fig. 4A). No similar survival advantage was seen when A431 epidermoid cancer cells (Fig. 4B), which express neither MSLN nor CA125, were inoculated intraperitoneally. Tumor growth was also assessed when the A549 lung cancer cells were inoculated subcutaneously. In this model, the tumors in *Msln*–*/*– and in *Msln+/+* mice grew at the same rate (Fig. 4C), demonstrating that *Msln* expression has no effect on tumor growth within this compartment.

**Table 1:** Effects of mMSLN-rFc, MPF-rFc and CD22-rFc on A549 cell growth in *Msln*(–/–) mice.

Experiment	Number ofMice	Median Survival(Days)	Percent of MiceSurviving >4Months	*P* Values
*Msln*(–/–)/PBS	41	46	24	-
*Msln*(–/–)/MPF-rFc	21	31	0	<0.0001
*Msln*(–/–)/CD22-rFc	21	46	10	0.388
*Msln*(–/–)/mMSLN-rFc	20	42	0	0.016

**Figure 5:**
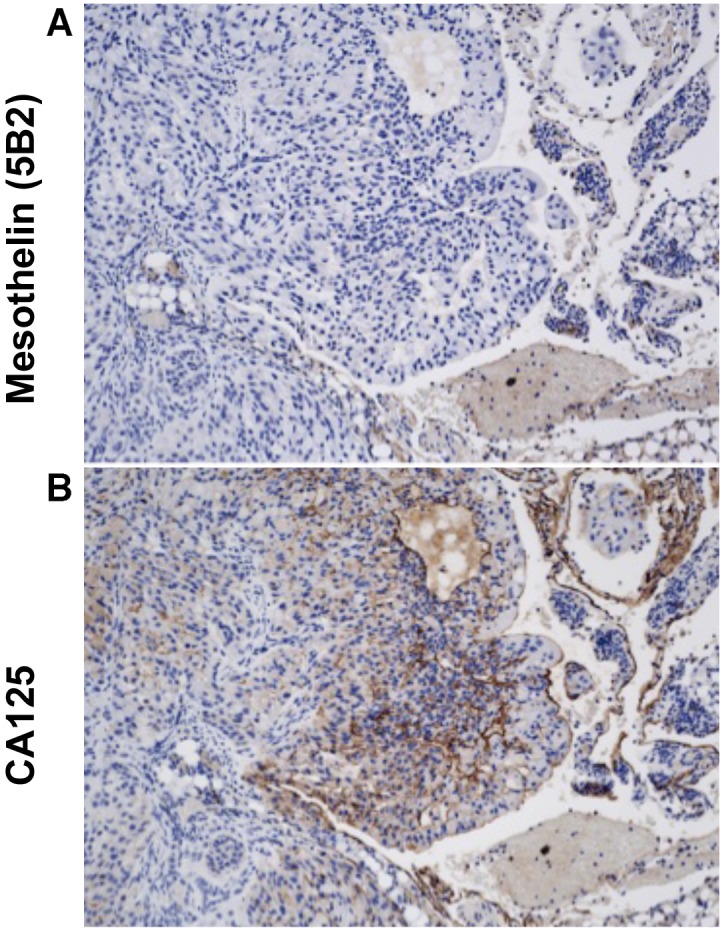
IP mouse model of A549 cells. An athymic nude mouse was sacrificed 26 days after IP inoculation with A549 cells. Tumors were immunostained with (A) anti-MSLN and (B) anti-CA125 monoclonal antibodies. These tumors were negative for MSLN but positive for CA125 expression as shown by brown staining of tumor cells.

In the intraperitoneal model, the abdomens of A549 tumor-bearing mice became swollen just before death. At necropsy, tumor masses were growing on the peritoneal wall, on the omentum, and on the large and small intestine. IHC demonstrates that these A549 tumors make no detectable MSLN (Fig. 5A), but do make CA125 (Fig. 5B). These results indicate that MPF, mMSLN or both can enhance the aggressiveness of A549 tumors within the abdominal cavity.

We hypothesized that if loss of *Msln* were responsible for the slowed tumor progression in the *Msln*–/– mice, then exogenous replacement of its protein products, MPF or mMSLN, might restore the more aggressive course of disease seen in the *Msln*(+/+) mice. Therefore, we synthesized recombinant MPF-rFc, mMSLN-rFc, and a control CD22-rFc protein. The strategic addition of the rabbit Fc moiety significantly increases the half-life of these molecules, but is not expected to interfere with function. A549 cells were injected into the peritoneal cavities of *Msln*–/– mice as before, then, beginning on the day of implantation, the mice were treated with one of the recombinant Fc proteins every other day for a total of six doses. As summarized in Table 1 and shown in Figure 4D, the median survival of *Msln*–/– mice treated with the CD22-rFc control peptide remained 46 days. However, median survival of mice treated with MPF-rFc decreased to 31 days, similar to the survival of *Msln*(+/+) mice injected with A549 cells (Fig. 4A). Injection of mMSLN-rFc also produced a statistically significant decrease in median survival, although the effect was less marked (42 days, p<0.016). The results of this experiment suggest that loss of Mpf rather than mMsln is primarily responsible for the prolonged survival of *Msln*–/– mice bearing xenografted A549 cells.

To determine if MPF acts directly on the A549 cells to enhance their growth or aggressiveness, we stably transfected the *MPF* cDNA into A549 cells. MPF expression was assessed by modified ELISA assay of conditioned medium and quantity produced by 1×10^6^ cells was calculated based on a standard curve of recombinant MPF. While A549/MPF clones produced approximately 400 ng of MPF in 48 hours, no MPF was detected in the condition medium of A549 cells transfected with vector control (A549/pcDNA). We noted no obvious difference in the growth rate or morphology of the *MPF*-transfected A549 cells when compared with the vector-transfected control (data not shown). Anchorage-independent growth of these cells in soft agar was also assessed. As shown in Table 2 there is no statistical difference in colony formation between *MPF*-transfected A549 cells and the vector-transfected control line. These experiments suggest that MPF does not directly promote the growth of A549 cells.

**Table 2:** Colony formation of stably transfected A549 cells in soft agar.

Experiment	A549/pcDNA			A549/MPF			KB		
	Mean	SD	n	Mean	SD	n	Mean	SD	n
1	65.4	26.7	5	74.4	32.1	5	104.5	20.3	6
2	59.4	10.0	5	54.8	9.2	5	107.8	10.3	6
3	64.6	14.7	5	69.2	12.3	5	130.0	7.0	3
**Mean**	**63.1**	**3.3**	**3**	**66.1**	**10.2**	**3**	**114.1**	**13.9**	**3**

## Discussion

For our study, we created a mouse model that lacks the *Msln* gene and is permissive to the growth of human cancer cells. The A549 lung cancer cell line used to form IP tumors in our experiments makes only minimal amounts of mMSLN, such that all MPF and mMSLN must be supplied exogenously. We observed a statistically significant 15 day difference in survival between *Msln+/+* and *Msln*–/– mice bearing intraperitoneal A549 tumors. This finding was cell-line specific and, interestingly, not observed in a subcutaneous model. These results indicate that a product of the *Msln* gene plays an important role in promoting *in* *vivo* tumor growth and progression for some types of cancer cells growing within the peritoneal cavity.

MPF and mMSLN are both protein products made by expression of the *MSLN* gene. Both proteins are highly expressed by a number of solid tumor malignancies including mesothelioma, pancreatic and ovarian cancers [1], [4]. Several studies have investigated how changes in expression of the *MSLN* gene can alter tumor growth, progression or invasiveness to produce a more aggressive tumor phenotype. Overexpression of the *MSLN* gene was shown to enhance interleukin (IL)-6 signaling [11], and to confer resistance to tumor necrosis factor (TNF)-α mediated apoptosis in pancreatic cancer cell lines [20]. Ectopic expression of the *MSLN* gene in a human breast cancer cell line promoted cell survival and anchorage-independent growth *in* *vitro* [10]. Other studies have demonstrated a vital role for the *MSLN* gene in regulating growth and apoptosis via both p53-dependent and independent pathways in pancreatic cancer cells [12]. In all of these *in* *vitro* studies, the pro-tumorigenic effects of *MSLN* gene manipulation were presumed to be mediated by changes in expression of mMSLN, although the genetic manipulations in the experiments should produce similar alterations of MPF levels.

To separate contributions made by MPF or mMSLN to the phenotype we observed in our study, we supplied exogenous recombinant protein to the *Msln*–/– mice. We found that MPF supplementation decreased animal survival 15 days compared to mice treated with a control protein. While supplementation with mMSLN did decrease survival a statistically significant 4 days, it is clear that MPF is the more potent factor in our model.

MPF was originally identified as a growth factor that could promote colony formation of megakaryocytes when given in combination with IL-3 [5]. To date, no MPF receptor has been identified on megakaryocytes or any other cells. Neither have theories been put forward to explain why a protein normally expressed exclusively by mesothelial cells should have a primary function as a megakaryocyte stimulating factor. Interestingly, in our experiments, while MPF increased tumor aggressiveness *in* *vivo*, forced overexpression of MPF produced no growth advantage *in* *vitro.* This suggests that the effect of MPF on tumor aggressiveness is not mediated by a direct action of MPF on the tumor, but perhaps by working indirectly on other cells. Although it might be tempting to consider that MPF may have an immune effector function, our experiments were conducted in lymphocyte-deficient mice, suggesting that another mechanism is more likely to be responsible. Identification of the cell type(s) responsible for mediating this effect of MPF is beyond the scope of the current study.

Although our observations were made using a single cell type, the effect we observed on survival was profound and highly reproducible. Our data suggest that MPF could be an important mediator of tumor aggressiveness in the peritoneal cavity, and that inhibition or sequestration of MPF might be clinically useful in slowing the growth of some cancers that grow in the peritoneal cavity. Studies to test this hypothesis have been initiated.
